# Electrochemical Impedance Sensors for Monitoring Trace Amounts of NO_3_ in Selected Growing Media

**DOI:** 10.3390/s150717715

**Published:** 2015-07-21

**Authors:** Seyed Alireza Ghaffari, William-O. Caron, Mathilde Loubier, Charles-O. Normandeau, Jeff Viens, Mohammed S. Lamhamedi, Benoit Gosselin, Younes Messaddeq

**Affiliations:** 1 Department of Electrical and Computer Engineering, Laval University, Quebec, QC G1V 0A6, Canada; E-Mails: seyed-alireza.ghaffari.1@ulaval.ca (S.A.G.); Benoit.Gosselin@gel.ulaval.ca (B.G.); 2 Department of Chemistry, Laval University, Quebec, QC G1V 0A6, Canada; E-Mails: william-olivier.caron.1@ulaval.ca (W.-O.C.); mathilde.loubier.1@ulaval.ca (M.L.); charles-olivier.normandeau.1@ulaval.ca (C.-O.N.); 3 Centre for Optics, Photonics and Lasers (COPL), Laval University, Quebec, QC G1V 0A6, Canada; E-Mail: jfviens@copl.ulaval.ca; 4 Ministère des Forêts, de la Faune et des Parcs. Quebec, G1P 3W8, Canada; E-Mail: Mohammed.Lamhamedi@mffp.gouv.qc.ca; 5 JIRU Instituto de Quimica, Araraquara-SP 14800-060, Brazil

**Keywords:** electrochemical impedance spectroscopy, nitrate sensor, microelectronics

## Abstract

With the advent of smart cities and big data, precision agriculture allows the feeding of sensor data into online databases for continuous crop monitoring, production optimization, and data storage. This paper describes a low-cost, compact, and scalable nitrate sensor based on electrochemical impedance spectroscopy for monitoring trace amounts of NO_3_^−^ in selected growing media. The nitrate sensor can be integrated to conventional microelectronics to perform online nitrate sensing continuously over a wide concentration range from 0.1 ppm to 100 ppm, with a response time of about 1 min, and feed data into a database for storage and analysis. The paper describes the structural design, the Nyquist impedance response, the measurement sensitivity and accuracy, and the field testing of the nitrate sensor performed within tree nursery settings under ISO/IEC 17025 certifications.

## 1. Introduction

The advent of smart cities allows sensor owners to register and connect their devices to feed data into an online database for storage, and to allow developers connect to the database and build their own applications [1]. Interest in smart cities is motivated by major challenges pertaining to sustainable growth, including the growth of urban infrastructure and rising urban populations, which, amongst other things, are putting pressures on food production, water supplies, and the environment. The need to reduce costs and resource consumption, to minimize fertilizer usage in the environment, and to optimize crop production worldwide have motivated the development of low-cost, online digital sensor technologies for monitoring the concentration of ionic nutrients in agriculture, notably nitrate, phosphate, and potassium [2]. Nowadays, the detection and identification of nutrients still rely on conventional sampling laboratory techniques, which are costly, laborious, and not always suitable for real-time monitoring in farm or large-scale industry settings. Therefore, a research challenge in this field is focused on the need to develop rapid, reliable, specific, and sensitive methods to detect and monitor these nutrients cost-effectively [3,4], while large scale analysis implies improved miniaturization, reduction of analysis time and cost, and multi-ion detection [5].

Precision agriculture (PA) is a farming management concept based on observing, measuring and responding to field variability in crops [6]. Crop variability typically has both a spatial and temporal component which makes data acquisition, computational treatments, and statistical analysis ubiquitous. The objective of precision agriculture research pertains to the ability of optimizing returns on inputs while preserving resources (water, fertilizer, *etc.*). The practice is enabled significantly by the development of digital sensor technologies arrayed across the field that communicate in real-time to a central management system the amount of phosphate, nitrate, or potassium in the soil or in other growing media. The system's ability to determine and locate in real-time the precise nutrient content allows for the creation of maps of the spatial/temporal variability of nutrient for specific crop management, which may be specific to open farm lands, tree nurseries, or greenhouse settings, *etc.* Measuring the spatial and temporal variables of crop using an array of nutrient sensors is key to defining smart strategies for efficient resource use and sustainable growth.

Many ion sensors or different methods have been developed in recent years to perform crop monitoring. Among these, Electrical Conductivity (EC) meters have been used extensively to measure soil salinity [7], however the lack of ion selectivity for this method makes it inadequate for the quantitative measurement of specific ions. In addition, EC measurement techniques such as Time Domain Reflectometry (TDR) and Frequency Domain Reflectometry (FDR) relate to the propagation of a voltage pulse and measurement of the reflected wave [8]; however, they are usually power hungry and processor intensive, which are sets of attributes inappropriate for low-cost sensors. Digital sensor technology based on electrical impedance spectroscopy (EIS) is becoming a powerful tool in precision agriculture because it involves a relatively simple electrical measurement that can readily be automated and whose results may often be correlated with many complex materials variables: from mass transport of fertilizers, rates of reactions with the growing medium, and local ion concentrations [9]. While EIS analysis has been used to perform tasks such as corrosion monitoring [10], fuel cell analysis [11], bio-sensing [12], mineral nutrient detection in plants [13,14], breast cancer detection [15], and glucose determination [16], this paper describes a novel, low-cost, and portable nitrate sensor based on EIS for the determination of trace amounts of NO_3_^−^ in selected growing media used in tree nurseries. The nitrate sensor can be integrated to conventional digital microelectronics or CMOS platforms to perform online nitrate sensing continuously, and feed data into a database for storage and analysis. This paper describes the structural design, the Nyquist impedance response, the measurement accuracy, and the field testing of the EIS nitrate sensor performed within a tree nursery setting under the International Organization for Standardization (ISO) and the International Electro-technical Commission (IEC) certifications #17025.

## 2. Chemistry of the EIS Sensor

### 2.1. EIS Nitrate Sensor Structure

**Figure 1:**
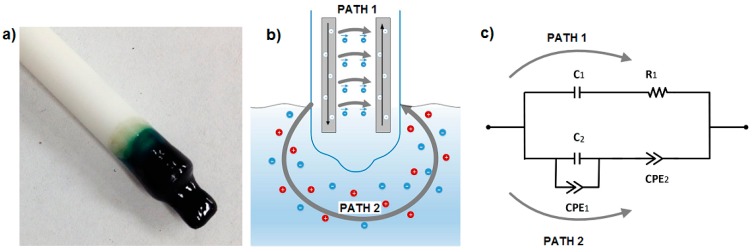
(**a**) Picture of the polyvinyl chloride-bis(2-ethylhexyl) phthalate (PVC-BEHP) electro-chemical nitrate sensor; (**b**) Schematics of the main electrical conduction paths, one within the polymer membrane and the other into the medium under test; and (**c**) Equivalent electrical circuit of the sensor.

The electrochemical nitrate sensor comprises a set of electrode wires surrounded by an ion selective polymer membrane, as shown in Figure 1a. The polymer membrane is inserted into the growing medium (preferably wet) and interacts locally with the medium under test. This sensor configuration provides two different electrical conduction paths, one within the polymer membrane and the other into the medium under test, depicted as paths 1 and 2 in Figure 1b, respectively. The equivalent electrical circuit of the sensor is described in Figure 1c and Section 3.4. The polymer membrane is composed of high molecular weight polyvinyl chloride (PVC—from Aldrich) and of a plasticizer bis(2-ethylhexyl) phthalate (BEHP—also from Aldrich). PVC-BEHP is an attractive scaffold for the development of low-cost, non-toxic, and chemically-stable sensors, and provides ease of fabrication and solubility in tetrahydrofuran (THF). Ion-selectivity is provided by adding two components to the polymer membrane: an ionophore and ionic sites. For the nitrate sensor, the ionophore consisted of tetramethyl cyclotetra-decanato-nickel(II) complex (NiTMTAA), and the ionic site consisted of trioctylmethylammonium chloride (TOMAC—from Aldrich). Both of these have been chosen according to the reversibility, selectivity (>4 $pK_{NO_{3}^{-},A^{-}}^{pot}$, where A^−^ stands for NO_2_^−^, HPO_4_^2−^, SO_4_^2−^, or Cl^−^) and efficiency reported in previous potentiometric studies [17]. All chemicals, except the synthetized ionophore, were reagent grade and used without further purification. Together, this polymer membrane composition can be dissolved into THF and molded into any desired shape prior to drying, which brings mechanical strength, environmental endurance, and abrasion resistance, and which defines a stable baseline of electrical conductivity to the system. The membrane exhibits low polarity in order to limit the entry of water and ions in the system. Also, the plasticizer reduces the glass transition temperature Tg of the polymer, and brings molecular mobility to the membrane; this property is necessary to enhance the speed of interaction with the medium under test.

The sensors were fabricated using a dip-coating process to cover the electrodes uniformly with the polymer membrane. The electrodes were made from copper wires (923UL-9 from Consolidated Electronic Wire and Cable), and inserted into a 5 mm-diameter 100 mm-long alumina rod comprising two parallel hollow cavities spaced by a gap of 0.6 mm. Alumina has been chosen for its dielectric, mechanical, and chemical strength properties. The copper wires protruded out of the alumina rod over a length of 5 mm along which the polymer membranes were coated. The dip-coating process consisted of immersing the protruding copper wires into a THF-dissolved polymer solution of abovementioned composition, followed by a 24-h drying at room temperature in order to let the THF evaporate and the polymer membrane solidify. Successive dipping and drying were performed until all the electrodes were fully covered by a 1 mm-thick membrane.

### 2.2. Medium under Test

Laboratory measurements were performed by immersing the PVC-BEHP electrochemical nitrate sensors in 10 mL of KNO_3_-containing (Aldrich—selectophore grade) deionized water (18 MΩ·cm) solutions set at 20 °C room temperature, while making sure to avoid sensor contact with any glassware surface. The sensors were left immersed in the solution for about 5 min to provide enough time for interaction and equilibrium with the ions. Field test measurements were performed in a white spruce (*Picea glauca* (Moench) Voss) seedling tree nursery setting. The measurement method consisted of immersing the sensors in about 300 mL of sampled growing medium (peat-vermiculite 80/20% v/v, density of 0.11 g/cm^3^, pH_H_2_O_ 3.8, pH_CaCl_2__ 3.1, C.E.C. 106 meq/100 g, N_min_ 53 mg/kg, N_NO3_ 6 mg/kg, P 13 mg/kg, K 20 mg/kg) mixed with water to reach saturation at about 92%wt of water content. The nitrate measurements obtained from the electrochemical sensors were compared with normalized colorimetric laboratory measurements, which consisted in sampling, centrifuging, and treating the growing medium using an ISO/IEC 17025-certified methodology described in Section 4.

## 3. Experimental Setup and Results

### 3.1. Measurement Setup

Impedance measurement and data collection for the PVC-BEHP electrochemical nitrate sensors were performed using a Solartron Impedance/Gain-phase Analyzer (model 1260A) through an AC frequency range from 1 Hz to 1 MHz. The Solartron measurements exhibited less than 5% error in the Real part of impedance and less than 1.5% in the Imaginary part of impedance, in the AC frequency range from 1 Hz to 1 MHz, and in the nitrate concentration range from 0 ppm to 6000 ppm. The AC amplitude of the driving signal was set at 200 mV to provide as low-signal, linear regime, and high S/N ratio as possible to the impedance measurements; however, the sensor exhibited impedance non-linearity as further described in Section 3.3. The connection of the Solartron Impedance analyzer to the sensor followed standard procedures described in Section 6.5 of the Solartron 1260 [18]. All the probes used during the tests were coaxial cables and the accuracy of the measurements was ±1% for the impedance magnitude and ±1 degree for phase. The measured impedance referred to the real and imaginary parts of the electrical impedance of the immersed sensors: *Z*(*ω*) = *R*(*ω*) + *jX*(*ω*), where *ω = 2πf*, *f* is the AC frequency of the measurement in Hertz, and *Z* is the compounded sum of resistances *R* and reactances *X* of the immersed sensor. The modulus of impedance is given by ||*Z*(*ω*)|| = $\sqrt{R{\left(\omega \right)}^{2}+X{\left(\omega \right)}^{2}}$ and the phase is given by ≮ *Z*(*ω*) = $\tan^{-1}(\frac{X(\omega )}{R(\omega )})$. Studying the impedance spectra of the system through a wide range of AC frequencies provides an objective and quantitative tool to assess medium variables such as nitrate ion concentration and water content, which may relate to specific elements of an equivalent circuit model for quick processing into useful data [9,19].

### 3.2. Measurement Results

**Figure 2:**
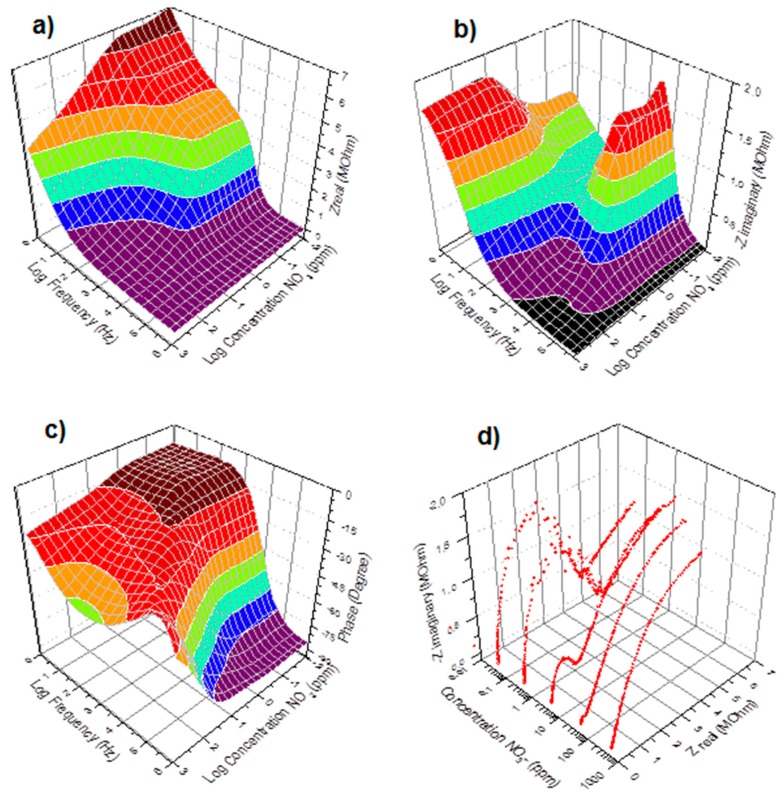
Real (**a**), Imaginary (**b**), Phase (**c**), and Nyquist (**d**) impedance spectra of the immersed PVC-BEHP electrochemical nitrate sensors, at 200 mV AC amplitude, through a wide range of nitrate (NO_3_**^−^**) concentrations using KNO_3_-containing water solutions.

Figure 2 shows the impedance spectra results of the immersed PVC-BEHP electrochemical nitrate sensors through a wide range of nitrate (NO_3_**^−^**) concentrations, under laboratory conditions using KNO_3_-containing water solutions. Measurement stability was reached after ~1 min immersion. These results show that the impedance spectrum of the PVC-BEHP sensor was strongly dependent on nitrate concentration throughout the range 0.01 ppm to 1000 ppm. The measured Nyquist profiles at the range of AC frequencies from 1 Hz to 1 MHz are indicative of a resistance-capacitance parallel circuit behavior that comprises phenomenological constant phase elements (CPE) as depicted in Figure 1c. A CPE is a component that models impedance elements exhibiting distributed materials properties, and has been proposed to explain the behavior of ionic charges in ionic conductors [20]. A conductivity dominated by the polymer membrane (Path 1) is key to obtaining selective nitrate sensor response; this membrane-related impedance was dominant at high frequencies and low NO_3_**^−^** concentrations, while the impedance of the medium (Path 2) became prominent at low frequencies and high (>100 ppm) NO_3_**^−^** concentrations, which leads to a non-selective nitrate sensor response at these conditions.

### 3.3. Measurement Non-Linearity

**Figure 3:**
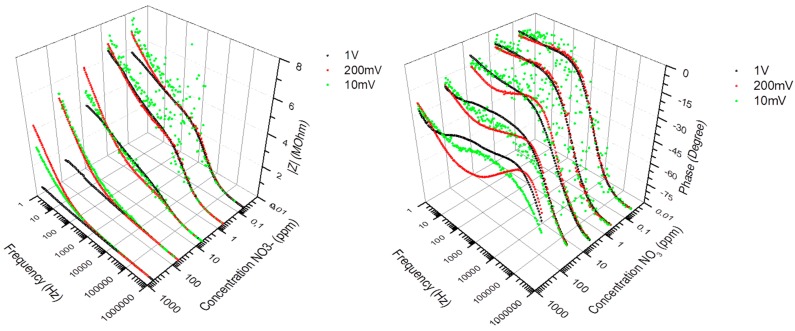
Impedance spectra (Modulus and Phase) of the PVC-BEHP electro-chemical nitrate sensors, at 10 mV (green dots), 200 mV (red dots), and 1 V (black dots) AC amplitudes, showing the measurement dependency with respect to the applied AC amplitude.

Electrochemical impedance spectroscopy involves the study of the variation of the impedance of an electrochemical system with the frequency of a small-amplitude AC perturbation. In practice, for the impedance measurement data to be reproducible, three main conditions have to be fulfilled. *Linearity*: the applied AC amplitude must be small enough so that the response of the system can be assumed to be linear, in first approximation, but still large enough to measure a response. Although highly reproducible under given conditions, the impedance spectra of the nitrate sensors did not fulfill the linearity assumption; Figure 3 shows that the recorded spectra exhibited a dependency with respect to the applied AC amplitude. The sensor had to be calibrated at a specific AC amplitude value, which was set at 200 mV with 0 V bias, as lower amplitudes led to poor S/N response and poor measurement accuracy. *Stability*: the overall state of the system must not change significantly during the acquisition of the data. This condition was fulfilled as the sensor exhibited a level of impedance stability of about ±5% during field tests in tree nursery settings, over a period of 1 month in air-slit containers of 25 square-shaped cavities (320 cm^3^/cavity, IPL 25-320, IPL Inc., Quebec, QC, Canada) filled with moistened 3:1 (v/v) peat-vermiculite. Over this period of time, the sensors were subjected to temperature fluctuations ranging from −2 °C to +29 °C in air, +1 °C to +23 °C inside the medium (obtained from thermistors), and to a cumulative irrigation of 65 mm (obtained from a rain gauge model No. TE525M, Texas Instruments). *Causality*: The measured AC response of the system must be directly correlated to the applied AC stimulus. This correlation implies selectivity to nitrate. Nitrate selectivity was not fulfilled when immersing the sensors in medium containing similar concentrations of Cl^−^ ions. As previously shown in Figure 2, the impedance of the medium (Path 2) becomes prominent at low frequencies and high (>100 ppm) NO_3_^−^ concentrations, which leads to a non-selective response of the sensors at these conditions. In order to alleviate ion cross-detection and achieve good measurement accuracies, this study used simple and controlled growing media such as substrates of peat moss and vermiculite, which relaxed selectivity requirements.

### 3.4. Equivalent Electrical Circuit

**Figure 4:**
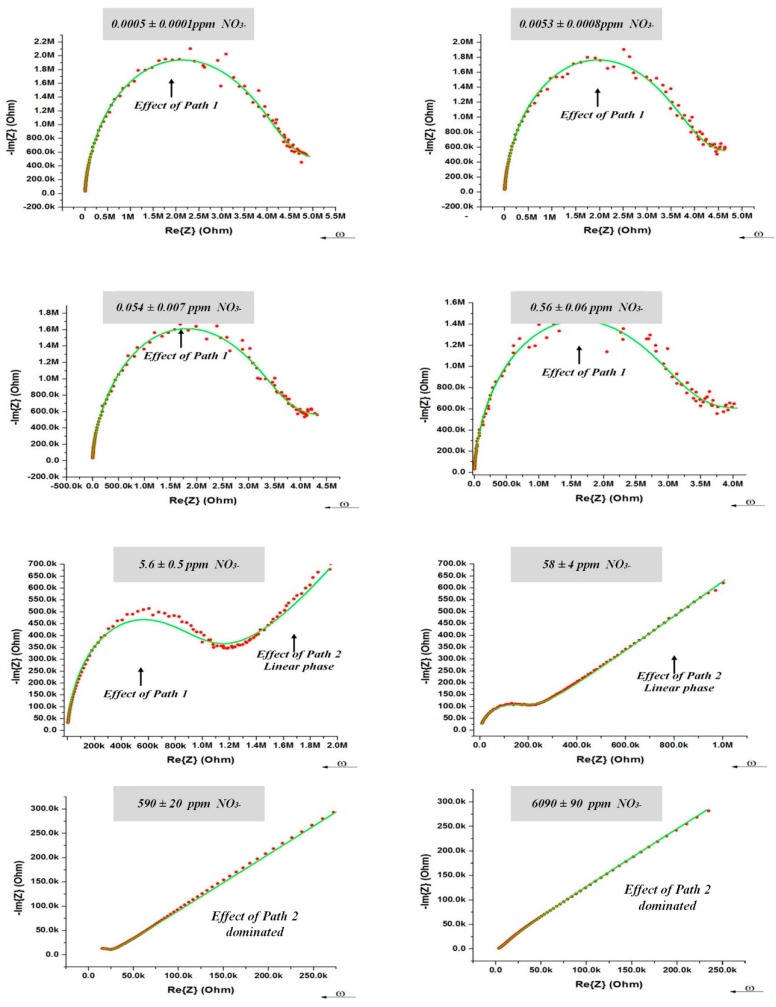
(Red dots) Nyquist response at 200 mV of the PVC-BEHP electro-chemical nitrate sensor through wide a range of nitrate (NO_3_**^−^**) concentrations. (Green lines) Fitting results using the equivalent electrical circuit model illustrated in Figure 1c.

Equivalent electrical circuits greatly aid in the process of fitting observed impedance data for elements with distributed properties. The equivalent electrical circuit model that represented the AC electrical conductivity of the PVC-BEHP electrochemical sensor is illustrated in Figure 1c. It is worth mentioning that both the PVC (path 1) and medium (path 2) are predominantly dielectric materials in nature and therefore exhibited poor DC electrical conductivities. Their electrical properties relate to various delocalized electrical carrier conduction mechanisms at play in non-crystalline materials such as short-range carrier mobility, defect hopping/trapping, and carrier diffusion, which manifest themselves negligibly in DC but may become significant and easily measured at AC frequencies in the kHz and MHz range [9,20,21]. In this context, the equivalent electrical circuit model contained both resistance and reactance (capacitive) elements which governed carrier mobility over a broad driving frequency range in AC. Specifically, the electrical circuit model assigned resistances and capacitances related to the nitrate-selective polymer membrane (Path 1: R_1_, C_1_) and to the non-selective medium under test (Path 2: C_2_-CPE_1_-CPE_2_) which comprised phenomenological constant phase elements (CPE). This electrical model could be applied accurately for all nitrate concentrations investigated in the present study, as demonstrated in Figure 4. The curve fitting had been done using an EIS analyzer software [22]. Overall, it can be seen that the electrical impedance of the polymer membrane was dominant at high AC frequencies and low nitrate concentrations, whereas the electrical impedance of the medium became dominant at low AC frequencies and high nitrate concentrations. As expected, the polymer membrane exhibited a negative phase in the impedance spectra indicative of a resistance-capacitance material response (Path 1), which, however, became strongly attenuated by the non-selective impedance of the medium at NO_3_**^−^** concentrations higher than 100 ppm (Path 2).

### 3.5. Sensor Circuit Miniaturization

**Figure 5:**
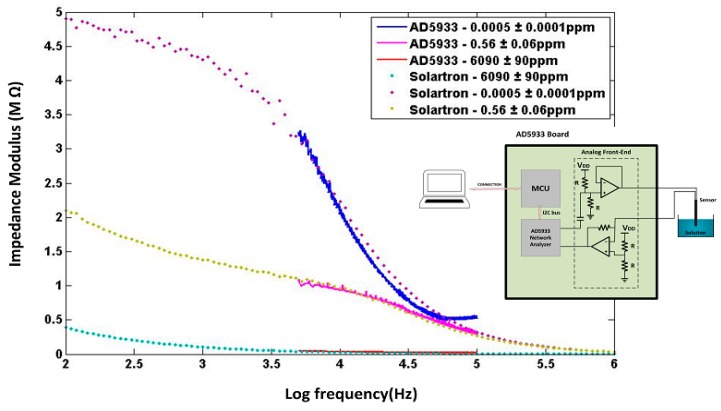
Comparative sensor impedance measurements made between the AD5933 microelectronics platform and the Solartron Impedance Analyzer.

An AD5933 microelectronics platform from Analog Devices was used to obtain a low-cost (~$30), compact (~8 × 8 cm^2^), and portable (~10 g) digital sensor technology that could be deployed, connected, and multiplexed at large scale during field tests. The AD5933 platform is a precision impedance converter circuit board that uses an auto-balancing method [23,24] for impedance measurements up to 10 MΩ, and that combines an AC frequency generator from 5 kHz to 100 kHz, with a 12-bit analog-to-digital converter (ADC). The frequency generator allows an external complex impedance (*i.e.*, sensor) to be excited with a known frequency. The impedance response signal was sampled by the ADC, a discrete Fourier transform (DFT) was processed by the on-board engine, and the results were sent to an online computer for data storage and analysis. The AD5933 employs a trans-impedance amplifier for amplifying the current and then uses the 1024 point on-chip DFT algorithm for separating the real and imaginary parts of impedance. After digitalizing with the ADC [25], a discrete-time impedance wave, Z[n] (*n* = 0, 1, 2, …N−1), is obtained by DFT:

$$F\{z[k]\}=Z(e^{j\omega })=\sum_{k=0}^{N-1}\text{z}[\text{k}].e^{\frac{-j2\pi kn}{N}}=\sum_{n=0}^{N-1}z[k]\cos(\frac{-j2\pi kn}{N})-j\sum_{n=0}^{N-1}z[\text{k}]\text{sin}(\frac{-j2\pi kn}{N})=Z_{i}-jZ_{q}$$

where Z*_i_* and Z*_q_* indicate respectively the real and imaginary parts of impedance returned by the DFT, both of which could be calibrated with respect to NO_3_ concentration in the medium at a specific AC amplitude, with best calibration resolution usually obtained when using the impedance modulus. To our knowledge, this is the first time the AD5933 device was used along with a chemical sensor for environmental analysis; for sake of carefulness this warranted comparison with other known and well calibrated impedance meters. As shown in Figure 5, the AD5933 impedance measurements have been compared with the Solartron impedance analyzer under the same laboratory conditions as described previously, but under a range of frequency limited from 5 kHz to 100 kHz according to restrictions determined by the oscillator module within the AD5933 device [26]. The AD5933 device exhibited higher measurement errors when operating close to upper or lower frequency limits of 5 kHz and 100 kHz, or far from the 200 kΩ internal calibration resistance. Overall, good agreement in impedance measurements was obtained between the AD5933 and Solartron, to within a ±10% comparative error level, through wide a range of nitrate concentrations. The comparative errors were about ±2% at 200 mV AC amplitude between 0.1 ppm and 100 ppm nitrate, which was the useful measurement range of field tests in tree nursery settings.

## 4. Field Testing in Tree Nursery Under ISO/IEC 17025 Certifications

**Figure 6:**
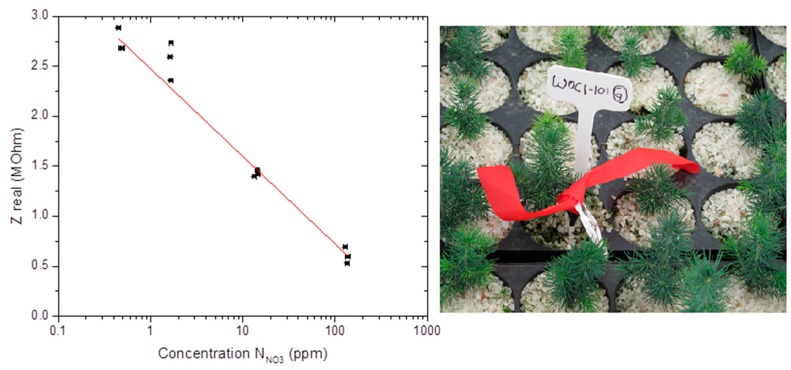
Field test results performed in growing medium selected from a white spruce tree nursery, showing the real part of the measured sensor impedance (in MΩ) at 1 kHz AC frequency compared against the ISO/IEC 17025-certified colorimetric NO_3_**^−^** concentration measurements.

Ppm-level measurement sensitivity and accuracy, environmental reliability and non-toxicity, and compatibility to ISO certifications are attributes sought for defining smart strategies for efficient and sustainable management of farm lands, tree nurseries, or greenhouses. For the nitrate sensors under study, field test measurements were performed in growing medium selected from a tree nursery of white spruce (*Picea glauca* [Moench] Voss) species of evergreen coniferous [27,28], shown in Figure 6. The sensors were inserted into the growing medium and could be used and calibrated under different nursery conditions, such as (1) *in-situ* under low irrigation conditions (~50%wt water); (2) *in-situ* after irrigation (~75%wt water); or (3) after sampling under water saturation (~90%wt water). For this study, an ISO/IEC 17025-certified methodology was adopted [29]. The measurement method consisted of immersing the sensors in about 300 mL of sampled growing medium (peat-vermiculite 80%/20% v/v, density of 0.11 g/cm^3^, pH_H_2_O_ 3.8, pH_CaCl_2__ 3.1) in a 600 mL beaker; this growing medium was then mixed with deionized water to reach saturation at about 92%wt of water content. The nitrate measurements obtained from the electrochemical sensor were made at that point, and later compared with normalized UV-V is colorimetric laboratory measurements taken of the same growing medium. The colorimetric measurements consisted of filtrating and centrifuging three samples of growing media, passing the aqueous centrifuged filtrate through a reductive column of copper-coated cadmium, and mixing with sulfanilamide and N-(1-naphtyl)ethylenediamine dihydrochlorid (NED). The reduction changed nitrate to nitrite which reacted with sulfanilamide, forming a diazonium compound that gave purple coloration with NED, yielding a specific optical absorption at 520 nm calibrated to provide a precise nitrate concentration. Figure 6 shows the sensor results (made after ~1 min measurement time in the sampled peat-vermiculite medium) compared to the UV-Vis colorimetric laboratory measurements of the filtrates (made after ~1 h of filtration, centrifugation, and reduction processes). The sensor results are indicative of ppm-level measurement sensitivity and accuracy for nitrate detection, with much improved ease, versatility, and economy as compared to conventional sampling laboratory techniques. The real part of the measured sensor impedance provided an accurate correlation with ISO/IEC 17025 certified measurements, in the tested range from 0.4 ppm to 130 ppm of NO_3_^−^ concentration, to within a measurement accuracy of about ±1 ppm (95% confidence level) in the range 1–10 ppm, and about ±10 ppm in the range 50–100 ppm.

## 5. Conclusions

With the advent of smart cities, precision agriculture allows the feeding of sensor data into online databases for continuous crop monitoring, production optimization, cost reduction, and data storage. This paper described a low-cost, compact, and scalable nitrate sensor based on electrochemical impedance spectroscopy for the determination of trace amounts of NO_3_**^−^** in selected growing media. The nitrate sensor can be integrated to conventional microelectronics or CMOS platforms to perform online nitrate sensing continuously over a wide concentration range from 0.1 ppm to 100 ppm, with a response time of about 1 min, and feed data into a database for storage and analysis. The paper described the structural design and microelectronics scaling, the ppm-level measurement sensitivity and accuracy, and the reliable field testing of the nitrate sensors performed within a tree nursery setting under ISO/IEC 17025 certifications, which were attributes sought for such sensors in defining smart strategies for efficient and sustainable management of farm lands, tree nurseries, or greenhouses.
